# Individual variability of olfactory fMRI in normosmia and olfactory dysfunction

**DOI:** 10.1007/s00405-020-06233-y

**Published:** 2020-08-14

**Authors:** Zang Yunpeng, Pengfei Han, Akshita Joshi, Thomas Hummel

**Affiliations:** 1 Department of Otorhinolaryngology, Interdisciplinary Center Smell and Taste, Medical Faculty Carl Gustav Carus, TU Dresden, Fetscherstrasse 74, 01307 Dresden, Germany; 2 Department of Otorhinolaryngology, The Affiliated Hospital XuZhou Medical University, XuZhou, People’s Republic of China; 3 Faculty of Psychology, Southwest University, Chongqing, People’s Republic of China

**Keywords:** fMRI, Olfactory dysfunction, Individual variability, Objective diagnosis

## Abstract

**Purpose:**

The diagnosis of olfactory dysfunction is mainly based on psychophysical measurements. The aim of the current study was to investigate how well the olfactory functional magnetic resonance imaging (fMRI) can effectively distinguish between normosmic people and subjects with olfactory dysfunction.

**Methods:**

Thirty-eight participants were recruited for the study. Group 1 consisted of 22 subjects with olfactory dysfunction (mean age = 44.3 years, SD = 18.6), and Group two consisted of 16 participants with normal olfactory function (mean age = 49.6 years, SD = 11.6). Olfactory functions were assessed in great detail for all participants, and brain activation in response to odorous stimulation was assessed using fMRI.

**Results:**

The between-group comparison showed stronger odor induced brain activation of the primary olfactory area and the insular cortex among the normosmic group as compared to the dysosmic group. As indicated by the individual analysis, positive responses in the primary olfactory cortex were significantly higher in normosmic people (94%) than in subjects with olfactory dysfunction (41%). However, there was no association between individual fMRI parameters (including the percentage of BOLD signal change, activated cluster size and peak *z* value), and psychophysical olfactory test scores. Receiver operating characteristic analysis suggested the subjects could not be differentiated from normosmics based on their BOLD signal from the primary olfactory area, orbitofrontal cortex, or the insular cortex.

**Conclusion:**

There are large inter-individual variabilities for odor-induced brain activation among normosmic subjects and subjects with olfactory dysfunction, due to this variation, at present it appears problematic to diagnose olfactory dysfunction on an individual level using fMRI.

## Introduction

Olfactory disorders are common with about one-fifth of the general population exhibiting decreased olfactory acuity and approximately 5% showing anosmia [1]. Olfactory dysfunction also can be seen as an early sign of neurodegenerative diseases such as Alzheimer's or Parkinson's disease [2] . Hence, detection of olfactory dysfunction receives an increasing amount of interest.

Overall, psychophysical tests including odor detection threshold, odor discrimination, and odor identification are widely distributed [3] While they are of high significance in the clinical routine, many factors may affect the reliability and repeatability of the test, such as culture [4] and education [5] Electrophysiological tests of olfactory function are available but are typically restricted to highly specialized centers [1]

Functional magnetic resonance imaging (fMRI) offers a noninvasive approach to assess olfactory function in an “objective” way [6]. On the cortical level, odor information is first processed in the primary olfactory areas (POC) including the piriform cortex, entorhinal cortex and amygdala. Further, olfactory information is processed in the secondary olfactory areas including the orbitofrontal cortex (OFC), insula, anterior cingulate cortex (ACC), striatal areas, thalamus, superior temporal gyrus, and hippocampus [7–9]. Previous studies have confirmed on a group level that subjects with olfactory dysfunction had reduced brain activation to odor stimuli in POC, OFC, and insular cortex compared to normosmic groups with normal olfaction [10–12]. No research has been done specifically to look into olfactory fMRI on an individual level among subjects with olfactory dysfunction. Olfactory fMRI has not reached routine clinical applications in patients with olfactory dysfunction. Therefore, the present study set out to examine how well fMRI signals to odorous stimuli could discriminate between individuals with normal olfactory function and people with little or no olfactory function.

## Materials and methods

### Participants

Thirty-eight subjects participated (mean age 46.6 years; 21 females, males) in this study. The cohort was divided into two groups according to their olfactory function. Of those, 22 were subjects (11 females) with diagnosed olfactory dysfunction, which was confirmed followed a standardized method [13] including standardized psychophysical testing of olfactory threshold, identification, and discrimination with the “Sniffin’ Sticks” test battery [14] patients’ characteristics were listed in Table 1. The rest of the participants consisted of 16 normosmic subjects (10 females). A detailed medical history was taken including conditions that potentially may have affected olfaction such as significant head trauma, chronic rhino sinusitis, neurological/endocrinological disorders, and previous nasal surgery. All included participants were in good health. Prior to inclusion in the study they received an otorhinolaryngological examination including nasal endoscopy. All participants were informed of the testing procedures and signed written informed consent before the experiment. The study design was in accordance with the Declaration of Helsinki and had been approved by the Ethics Committee of the Medical Faculty Carl Gustav Carus at the Technical University of Dresden.

**Table 1:** Patients' characteristics

	No	Overall olfactory function	Duration of olfactory loss [months]	Age [years]	Sex
		(TDI score)			
Congenital olfactory loss (*n* = 14)	P01	13		67	Female
	P02	11		64	Male
	P03	12.5		48	Female
	P04	14		23	Male
	P05	17.5		19	Male
	P06	12		34	Male
	P07	11		27	Female
	P08	7.25		30	Male
	P09	13		30	Female
	P10	16		39	Female
	P11	12		23	Male
	P12	10		76	Female
	P13	9		22	Male
	P14	15.25		22	Female
Idiopathic olfactory dysfunction (*n* = 8)	P15	10	9	33	Female
	P16	18.25	12	64	Female
	P17	14	108	57	Male
	P18	11	48	54	Female
	P19	6.25	48	70	Male
	P20	1	96	55	Female
	P21	18	36	58	Male
	P22	9	84	60	Male

### Assessment of olfactory function

All participants received birhinal olfactory testing using the validated and reliable "Sniffin' Sticks" battery (Burghart, Wedel, Germany). The test consists of measurements of odor sensitivity (phenyl ethyl alcohol odor detection thresholds), odor discrimination, and odor identification [14] First, odor threshold (*T*) is assessed using phenyl ethyl alcohol (a rose-like odor) presented by means of a single staircase, using stepwise dilutions in a row of 16 felt tip pens. Second, odor discrimination (*D*) is assessed by asking subjects to perform a three-alternative forced choice task using 16 pairs of odors. Third, odor identification (*I*) is assessed by asking subjects to identify 16 individual odors from a list of four verbal descriptors using a forced choice task. Scores from threshold (*T*), discrimination (*D*) and identification (*I*) were then added up to provide the TDI score The TDI score was the base for the diagnosis of olfactory dysfunction as well as its severity (e.g. hyposmia or anosmia). The TDI score ranges from 1 to 48, with a score above 30.5 being considered as normosmia, TDI score between 16 and 30.5 as hyposmia, and TDI score less than 16 as functional anosmia [15,16]

### Odor stimulation paradigm

One odor was used as stimuli: “Coffee” odor (Frey und Lau, Henstedt-Ulzburg, Germany). Odorless air served as control stimulus. A block design was adopted for odor stimulation during fMRI measurements. During each functional run, each block lasted for 20 s, during one block, odorized air and was delivered to the participants’ bilateral nostril intermittently (8 s for odorized air, followed by 12 s of odorless air), in order to minimize adaptation to the odors which refers to a recent literature indicating larger BOLD signal in response to odor stimulation in shorter runs compared to longer but fewer runs [17], totally 12 blocks and lasting 240 s. Odorized and odorless air were delivered at a flow of 2l/min using a portable olfactometer.

### MRI data acquisition and preprocessing

A 3 T MRI scanner (Siemens Prisma, Erlangen, Germany) with a 32-channel head coil was used for image acquisition. For functional image collection, a spin echo/echo planar imaging (EPI) sequence was applied with echo time (TE) = 30 ms, repetition time (TR) = 2000 ms, flip angle = 90°, and in-plan resolution 2 mm. A T1-weighted structural scan was collected after functional runs with 121 slices, voxel size 1 × 1 × 1 mm^3^.

Imaging data were analyzed by means of SPM12 (Wellcome Trust Centre for Neuroimaging, London, UK) implemented in Matlab (version 2013a, Mathworks, Natick, MA, USA). Functional image volumes were pre-processed, starting with realignment and unwarp. In addition, the high-resolution T1 image was co-registered to the mean image of the EPI series for each participant. Co-registered T1-weighted MR images were segmented for gray and white matter to compute spatial transformation parameters for normalization. The registered functional scans were normalized to a standard Montreal Neurological Institute (MNI) template. Normalized images were spatially smoothed with an 8-mm full-width half-maximum Gaussian kernel. Finally, removal of head motion artifacts using ArtRepair (version 4, Stanford University) was applied to the preprocessed images based on the following rules: image to image motion less than 0.5 mm/TR and total images repaired less than 20%.

### MRI data analysis

**Fig. 1:**
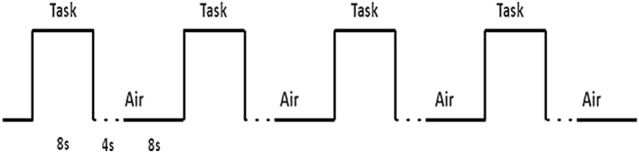
fMRI block design paradigm, totally 12 times of repeated “Task-Air” cycles. The time for each task is 8 s, the total time for presenting air is 12 s, during the odorless presentation, the first 4 s are buffer time

**Fig. 2:**
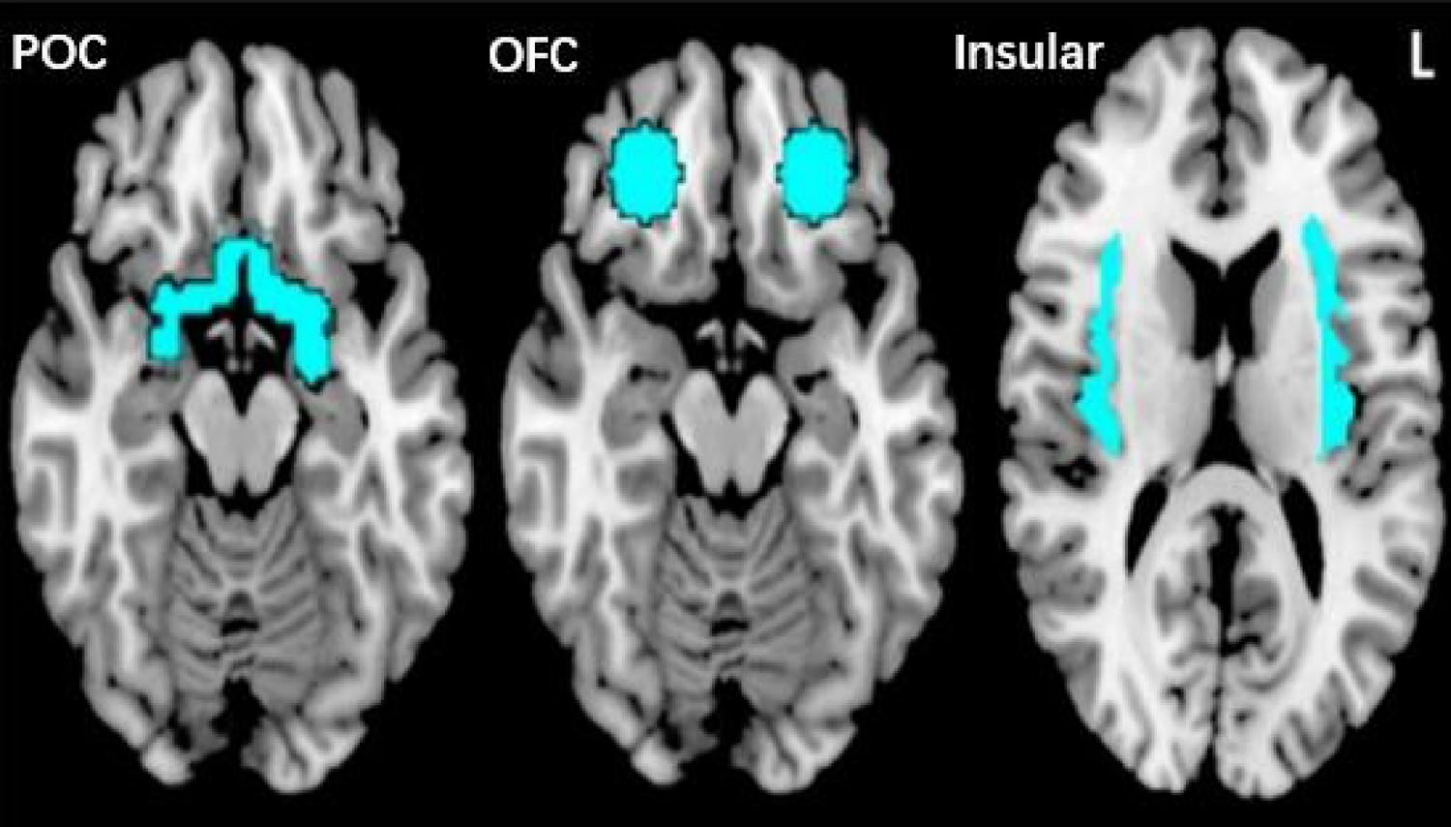
The light blue regions stand for the selected ROIs template: POC, OFC and Insular

First, MRI scans were checked by an experienced radiologist for possible pathological brain anomalies. On the first level, the contrast “odor ON vs odor OFF” was modeled for each participant, in each block, we used the first 8 s coffee odor stimulation compared to the last 8 s odorless air (Fig. 1). The 1st level contrasts were submitted into second level analysis to show: (1) brain activation in patients and normosmic groups separately, and (2) different brain activation between the two groups. Analyses were focused on three regions of interest (ROIs): the primary olfactory cortex (POC), the olfactory orbitofrontal cortex and the insular cortex. The anatomical mask defined by Fjaeldstad et al.[8] including the piriform cortex and part of the amygdala, was used as the mask for POC. The olfactory OFC mask was built based on a 10-mm sphere centered on the right (*x*, *y*, *z*: 24, 36, −12) or left putative olfactory OFC (*x*, *y*, *z*: −22,32,−16) [17]. The insular cortex ROI was defined with the WFU_PickAtlas software (ANSIR, Wake Forest University, Winston-Salem, NC, USA)[18] . The template of the region for each ROI was showed in Fig. 2.

On an individual level, we conducted anatomical region-of-interest (ROI) analyses by extracting the mean % BOLD signal change (% BOLD signal change is often calculated using a baseline of the mean of the time series on a voxel, the MarsBaR software will calculate a baseline from the mean signal over a small ROI of many voxels) for each participant from all voxels in three ROIs [19] The anatomical ROI approach increases statistical power by averaging BOLD activation across multiple voxels within a specific brain region using the MarsBaR toolbox [20]. thereby reducing noise and the need to correct for multiple comparisons. Critically, anatomical ROIs are independent of the functional data analyzed, resulting in unbiased estimates of effect size [21].

Next, the spatial distribution of activation extent (defined as the number of significantly activated voxels) and intensity (defined as the cluster peak *z* value) were also extracted for individual participants from each ROI. An activation was considered significant when surviving to an uncorrected threshold of *p* < 0.05. The overall visualization and screenshots were performed using the software of MRIcroGL (https://www.cabiatl.com/mricrogl/). The receiver operating characteristic (ROC) curve was constructed to test the ability of the % BOLD signal change within each of the three ROIs to discriminate between people with normal olfaction and patient with olfactory dysfunctions, defined based on their TDI scores. The classification accuracy was quantified using the area under the (AUC) value. All statistical analysis was performed by means of SPSS 23 (SPSS Inc., Chicago, IL, USA). Pearson correlation was performed between activations response of odor stimulation of selected ROIs of individuals and the Sniffin’ Sticks test scores. Chi-square analysis was used in calculating the proportion of effective activations response of odor stimulation of selected ROIs of individuals between normosmics and subjects with olfactory dysfunction.

## Results

### Group level brain activation

**Fig. 3:**
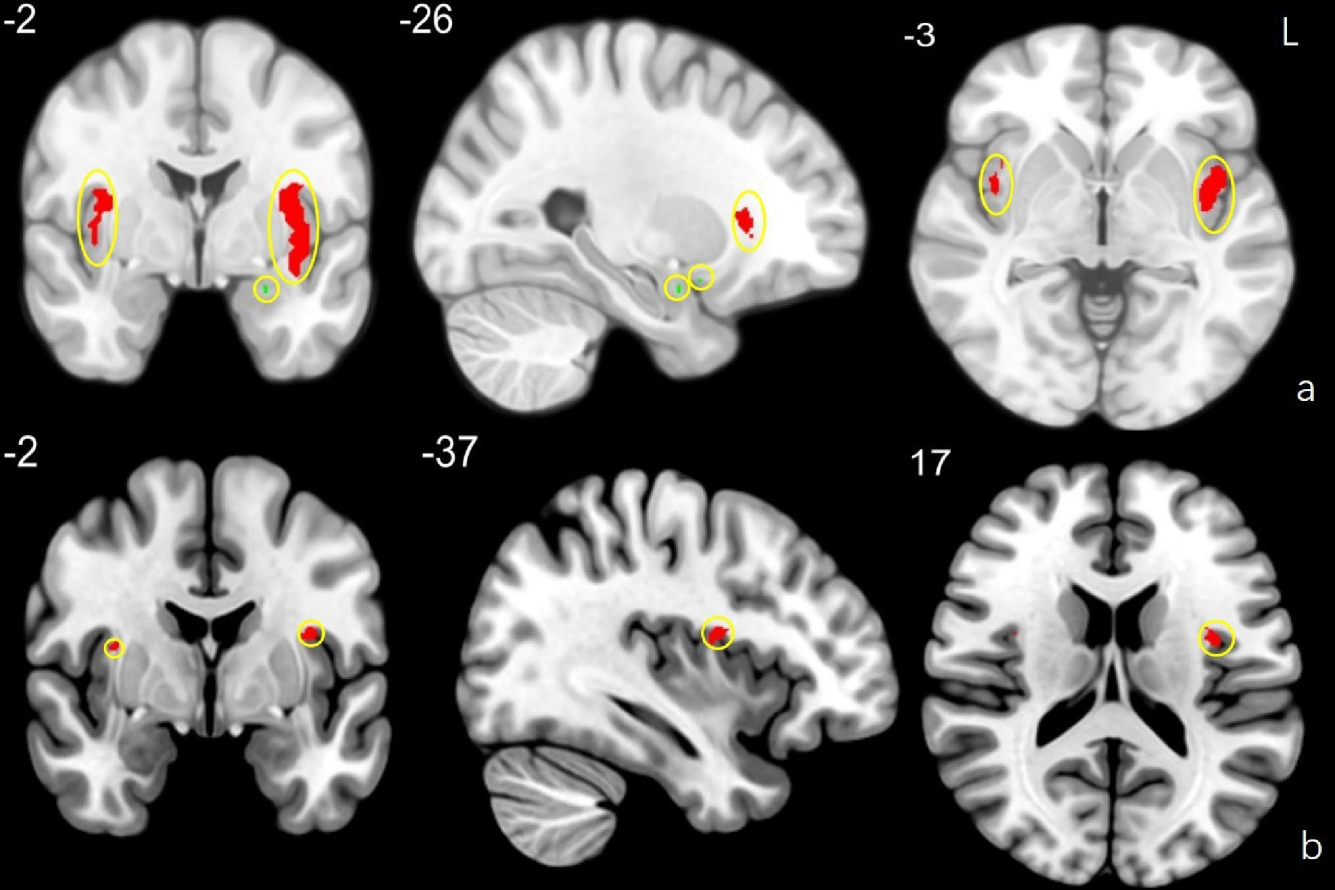
**(a)** fMRI in 3 dimensions from a normosmic subject, red region indicates the activated voxel clusters in the insular, green region indicates the activated voxel clusters in the POC. (**b**) fMRI in 3 dimensions from a subject with olfactory dysfunction, red region indicates the activated voxel clusters in the insular. Yellow circle is used to emphasize the activated voxel clusters

Brain activations to odor stimulation in the normosmic group were shown in Fig. 3a, the local maxima of the main activated areas were found in the left POC (Montreal Neurological Institute [MNI] peak coordinates −26, −2, −18), insula (MNI peak coordinates −38, −8, 6 and 36, 0, 16), no obvious activations were found in the OFC (Fig. 3a). In subjects, the local maxima of the main activated areas were found in the left insula (MNI peak coordinates −36, −2, 18), no obvious activations were found in the POC and OFC regions (Fig. 3b). Between group comparison revealed stronger activation in the POC among normosmics as compared to subjects. Table 2 recaps maximum activation voxels and peak-*z* value responses to olfactory stimulation obtained from the the POC, OFC and insula regions in the group-level and found that the intensity of activation response to odor stimulation was much higher in the insular region than other ROIs.

**Table 2:** Data statistics for individuals, BOLD signal change obtained in POC, OFC, insular from each subject in response to odor stimulation

		TDI	Age (years)	Sex	ROI (signal change%)		
					POC	OFC	Insular
Subject with olfactory dysfunction (*n* = 22)	P01	13.00	67	f	0.08	0.05	0.03
	P02	11.00	64	m	0.04	0.11	0.00
	P03	12.50	48	m	0.06	− 0.05	0.01
	P04	14.00	23	m	− 0.20	− 0.11	− 0.09
	P05	17.50	19	m	0.10	− 0.05	− 0.02
	P06	12.00	34	m	− 0.05	− 0.04	− 0.04
	P07	11.00	27	f	0.17	0.10	0.29
	P08	7.25	30	m	0.02	0.08	− 0.07
	P09	13.00	30	f	0.14	0.30	− 0.01
	P10	10.00	33	f	0.00	− 0.02	− 0.16
	P11	16.00	39	f	− 0.05	0.20	0.56
	P12	18.25	64	f	0.00	− 0.01	− 0.07
	P13	14.00	57	m	0.11	0.13	− 0.06
	P14	12.00	23	m	− 0.26	− 0.16	− 0.28
	P15	11.00	54	f	− 0.02	− 0.06	− 0.05
	P16	6.25	70	m	− 0.21	− 0.06	− 0.25
	P17	1.00	55	f	0.01	0.11	0.20
	P18	10.00	76	f	0.07	− 0.05	− 0.17
	P19	18.00	58	m	− 0.03	0.09	0.03
	P20	9.00	22	m	− 0.11	0.02	− 0.09
	P21	15.25	22	m	− 0.16	− 0.03	0.02
	P22	9.00	60	m	− 0.04	0.05	− 0.03
Normosmia (*n* = 16)	C01	30.75	59	f	0.14	0.14	0.07
	C02	30.25	65	m	0.03	0.12	− 0.01
	C03	34.00	48	f	− 0.14	0.07	0.05
	C04	39.50	45	m	0.23	0.11	0.11
	C05	38.00	49	f	− 0.04	− 0.01	0.12
	C06	30.00	48	f	0.07	0.01	0.03
	C07	36.00	30	f	− 0.06	− 0.06	− 0.05
	C08	31.00	59	f	− 0.08	0.04	0.07
	C09	39.50	57	f	0.04	0.12	0.02
	C10	35.50	37	f	0.12	− 0.05	− 0.03
	C11	38.50	64	f	− 0.02	− 0.09	− 0.12
	C12	34.50	21	m	0.16	0.28	0.48
	C13	40.50	48	m	− 0.13	− 0.14	− 0.17
	C14	37.25	43	f	− 0.08	0.13	0.06
	C15	30.75	55	m	0.04	0.24	0.12
	C16	37.75	59	m	− 0.06	− 0.04	− 0.07

### Individual variability

Tables 3 and 4 show the % of BOLD signal change, cluster size (assessment of significant activation in fMRI) and peak-*z* value (normalized magnitude of peak voxel from statistical parametric map from publication) in responses to odor stimulation obtained from the three ROIs ROI for each participant. No significant correlation between TDI scores and the % of BOLD signal change, cluster size or peak-*z* value was found (*p* > 0.05).

**Table 3:** Cluster size, peak-*z* value obtained in POC, OFC, Insular from each subject in response to odor stimulation

		POC		OFC		Insular	
		Cluster size (voxel)	Peak-*z* score	Cluster size (voxel)	Peak-*z* score	Cluster size (voxel)	Peak-*z* score
Subject with olfactory dysfunction (*n* = 22)	P01	5	1.65	20	2.04	9	2.17
	P02	7	2.04	38	2.18	1	1.73
	P03		− 4.00	−	−	−	−
	P04	–	−	3	1.80	−	−
	P05	−	−	−	−	3	1.72
	P06	18	1.69	31	2.22	14	2.11
	P07	−	−	8	1.94	46	2.40
	P08	−	−	10	2.39	123	2.85
	P09	59	2.22	104	2.92	128	3.60
	P10	−	−	21	2.33	138	2.44
	P11	−	−	6	2.14	−	−
	P12	−	−	1	1.64	12	1.97
	P13	−	−	−	−	−	−
	P14	−	−	−	−	−	−
	P15	−	−	−	−	−	−
	P16	12	1.99	20	2.69	59	2.13
	P17	23	2.28	7	1.85	90	2.63
	P18	−	−	−	−	1	1.75
	P19	2	1.41	−	−	−	−
	P20	19	1.93	−	−	1	1.66
	P21	−	−	14	2.26	12	1.89
	P22	−	−	−	−	18	2.22
Normosmia (*n* = 16)	C01	1	0.01	1	1.89	4	1.82
	C02	16	1.85	27	2.14	36	2.26
	C03	8	1.84	−	−	4	2.20
	C04	−	−	−	−	−	−
	C05	114	3.16	164	3.25	1503	5.57
	C06	1	1.31	−	−	−	−
	C07	7	1.84	−	−	−	−
	C08	5	1.55	38	2.65	1	1.81
	C09	5	1.56	–	−	−	−
	C10	4	1.59	–	−	13	2.11
	C11	3	1.42	1	1.79	2	1.87
	C12	112	4.52	231	3.44	1443	5.12
	C13	2	1.43	31	2.46	−	−
	C14	2	1.41	72	2.65	3	1.98
	C15	1	1.35	60	2.54	473	3.76
	C16	12	2.34	10		−	−

–means no obvious activations obtained in this ROI

**Table 4:** Cluster size, peak-*z* value in response to odor stimulation obtained from POC, OFC and Insula ROIs in normosmic subjects, subjects with olfactory dysfunction and normosmic group against subjects with olfactory dysfunction

	*k*	*z*	*x*	*y*	*z*	ROIs
Normosmics	2	2.76	−26	−2	−18	POC
	–	−	−	−	−	OFC
	646	4.29	−38	−8	6	Insula
Subjects with olfactory dysfunction	−	−	−	−	−	POC
	−	−	−	−	−	OFC
	16	2	−36	−2	18	Insula
Normosmics > subject with olfactory dysfunction	3	2.12	−26	8	−16	POC
	−	−	−	−	−	OFC
	201	2.65	−36	−6	4	Insula

–means no obvious activations obtained in this ROI.

Table 4 summarizes the cluster size and peak *z*-value of each ROIs for each participant. With the applied threshold (uncorrected *p* < 0.05), 93.8%, 62.5%, 62.5% of the normosmics showed detectable cluster (minimum 1 voxel) in the POC, OFC or insula, respectively. In the dysosmic group, the percentage of participants with observed activated clusters was 40.9%, 59.1% and 68.2% for the POC, OFC and insula, respectively. The proportion of activated clusters in POC in normosmic participants (93.8%) was significantly higher than that in subjects with olfactory dysfunction (40.9%) (*X*^*2*^ = 14.9, *P* < 0.001) ROC analyses showed no significant AUC for % BOLD signal change for all three ROIs (POC: AUC = 0.53*,* *p* = 0.72; OFC: AUC = 0.58, *p* = 0.41; insula: AUC = 0.67, *p *= 0.07), meaning that it was not possible to distinguish between normosmics and subjects with olfactory dysfunction by means of the % BOLD signal change.

## Discussion

The present study explored the odor-induced brain activation at an individual level among subjects with olfactory dysfunction and normosmics with a normal sense of smell. Results suggest a large inter-individual variability, including the average BOLD signal in predicted ROIs, peak *z* value as well as the activated cluster size at a given threshold. While the between-group comparison revealed stronger activation among normosmics than subjects with olfactory dysfunction, subjects with olfactory loss could not be separated from normosmics according to their BOLD responses based on individual information. In other words, the BOLD signal on an individual level could not predict olfactory performance accurately, though the present study indicated that there is a significantly higher proportion of odor-induced activations in the POC of normosmics compared to subjects with olfactory dysfunction. Previous research has indicated high variability of olfactory fMRI among normosmics [22] . Our present results corroborate and extend these findings showing the variability in subjects with olfactory dysfunction.

The olfactory BOLD signal suffers from the magnetic artifact which decreases the signal-to-noise ratio (SNR), which in turn lowers the sensitivity to detect the BOLD signal in the olfactory system. Besides, physiological factors such as respiration and metabolic status also influence the sensitivity for detecting differences between groups. Some approaches have been suggested to increase SNR regarding the odor stimulation paradigm, e.g., short odor stimulation time [23,24] rapid repetition time (< 1 s) during brain image acquisition [25] or task protocols applied (e.g. synchronization of the breathing cycle with odor stimulation) [23] In addition, sequential stimulation with increasing intensities of odor may potentially offset potential habituation effects [26] Moreover, the presently used odors were familiar food-related. This association to food may constitute an angle through which the odor could be influenced by affective or metabolic factors. Unfamiliar neutral odors (e.g. “olfactory white” [27] may be a possible alternative choice.

The challenge of developing fMRI biomarkers for individual subjects is a general issue, due to the relatively low reliability of the fMRI signal [28,29]. As stated above, the individual difference measured with fMRI may also be attributed to the noise from the measurement [30]. Further, within-subject variance such as motion and physiological changes may also influence the stability of the measurement which additionally influences the weak olfactory signal with low signal-to-noise ratio. It has been suggested that precision functional mapping of individual human brains can be achieved with larger datasets [31].

One recent study demonstrated that odor-induced brain activation in the amygdala covaried with the participants’ odor sensitivity [23]. However, such correlation does not ensure the generalizability of the established relationship to out-of-sample individual subjects. Future research should aim to shift from such simple correlations to predictions. We may be able to interpret the fMRI-derived statistics of an individual subject in light of its distribution in a larger, normative sample. This approach could lead, for clinical imaging, to a more biologically informed science of human disease and a better basis for personalized treatment. Besides, with the advances in understanding the precise functional regions for olfactory processing and the higher resolution imaging technique, more precise location for the brain structures involved in initial processing of odor stimuli could be identified.

## Conclusion

The present study showed poor separation between individual normosmics and individual subjects with olfactory dysfunction based on odor-induced activation in key olfactory brain regions. Across the two groups the POC region showed more activation to odors in normosmic subjects compared to subjects with olfactory dysfunction. At present it appears problematic to diagnose olfactory dysfunction on an individual level using fMRI.
